# Patient caught breastfeeding and instructed to stop: an empirical ethics study on marijuana and lactation

**DOI:** 10.1186/s42238-022-00127-y

**Published:** 2022-04-12

**Authors:** Marielle S. Gross, Margot Le Neveu, Kara A. Milliken, Mary Catherine Beach

**Affiliations:** 1 Department of Obstetrics, Gynecology, and Reproductive, Sciences, University of Pittsburgh Medical Center, Magee Women’s Hospital, 300 Halket St, Pittsburgh, PA 15213 USA; 2 Department of Gynecology and Obstetrics, Johns Hopkins Hospital, 1800 Orleans St, Baltimore, MD 21287 USA; 3 David S. Olton Behavioral Biology Program, Zanvyl Krieger School of Arts and Sciences, Johns Hopkins University, 3400 N Charles St, Baltimore, MD 21218 USA; 4 Johns Hopkins Berman Institute of Bioethics, 1809 Ashland, Ave, Baltimore, MD 21205 USA

**Keywords:** Breastfeeding, Cannabis, Marijuana, Hospital policy, Stigma

## Abstract

**Background:**

The US guidelines recommend avoiding marijuana during breastfeeding given concerns about infant’s neurodevelopment. In this setting, some physicians and hospitals recommend against or prohibit breastfeeding when marijuana use is detected during pregnancy. However, breastfeeding is beneficial for infants and women, and stigmatization of substance use in pregnancy has been historically linked to punitive approaches with a disproportionate impact on minority populations. We advance an empirically informed ethical analysis of this issue.

**Methods:**

First, we performed a retrospective cross-sectional qualitative study of prenatal and postpartum records from a random sample of 150 women delivered in an academic hospital system in 2017 to provide evidence and context regarding breastfeeding management in relation to marijuana use. We then perform a scoping literature review on infant risks from breastmilk marijuana exposure and risks associated with not breastfeeding for infants and women. Finally, we analyze this issue vis-a-vis ethical principles of beneficence, autonomy, and justice.

**Results:**

(1) Medical records reveal punitive language pertaining to the medicinal use of marijuana in pregnancy and misinterpretation of national guidelines, e.g., “patient caught breastfeeding and instructed to stop.”

(2) Though there are plausible neurodevelopmental harms from breastmilk exposure to THC, evidence of infant effects from breastmilk exposure to marijuana is limited and largely confounded by concomitant pregnancy exposure and undisclosed exposures. By contrast, health benefits of breastfeeding for women and infants are well-established, as are harms of forgoing breastfeeding.

(3) Discouraging breastfeeding for women with marijuana use in pregnancy contradicts beneficence, as it neglects women’s health considerations and incorrectly assumes that risks exceed benefits for infants. Restrictive hospital practices (e.g., withholding lactation support) compromise maternal autonomy and exploit power asymmetry between birthing persons and institutions, particularly when compulsory toxicology screening prompts child welfare investigations. Finally, recommending against breastfeeding during prenatal care and imposing restrictions during postpartum hospitalization may exacerbate racial disparities in breastfeeding and related health outcomes.

**Conclusions:**

Policy interpretations which discourage rather than encourage breastfeeding among women who use of marijuana may cause net harm, compromise autonomy, and disproportionately threaten health and wellbeing of underserved women and infants.

## Background

The legalization of medical and recreational marijuana across the USA and the rising utilization of other cannabinoid derivatives has corresponded with evolving perceptions of safety, increasing potency, and widespread access to the once-illicit substance. Pregnant and breastfeeding populations are no exception. National surveillance data found roughly 5% of pregnant women reported use in the past month—over a 60% increase in the prior decade—with the highest rates among young, urban, and low-income individuals (National Survey on Drugs Use and Health n.d.; Crume et al. 2018; Ryan et al. 2018; Jarlenski et al. 2017). Data regarding the prevalence of marijuana use while breastfeeding is relatively scant, but appears consistent with the 5% rate in pregnancy (Crume et al. 2018).

The American Academy of Pediatrics (AAP) recommends breastfeeding and human milk as the normative standards for infant feeding given substantial evidence of health and psychosocial benefits (Policy Statement: breastfeeding and the use of human milk 2012). The US professional guidelines (Table 1) recommend avoiding marijuana during breastfeeding. Both the American College of Obstetricians and Gynecologists (ACOG) and the AAP acknowledge the paucity of data to assess the effects of marijuana exposure on infants during breastfeeding (Policy Statement: breastfeeding and the use of human milk 2012; Ryan et al. 2018; Marijuana Use During Pregnancy and Lactaqtion 2017). The Centers for Disease Control (CDC) acknowledges the risk of exposing the neonate to chemicals through breastfeeding, and the Academy of Breastfeeding Medicine cites concerns specifically regarding infants’ long-term neurobehavioral development (Reece-Stremtan and Marinelli 2015; Marijuana 2021). While it is difficult to isolate the effect of marijuana on infants through breastmilk alone, one systematic review of six studies found marijuana exposure in breastmilk to be associated with decreased motor development in infants at 1 year (Seabrook et al. 2017). In our experience, concerns about infant exposure to marijuana metabolites in breastmilk may prompt clinicians to discourage or prohibit breastfeeding for those whose marijuana use was detected in pregnancy.

**Table 1:** National guidelines for breastfeeding in the setting of marijuana

Organization	Representative quote summarizing policy
Centers for Disease Control and Prevention (Marijuana 2021)	Using marijuana while breastfeeding can allow harmful chemicals to pass from the mother to the infant through breastmilk or secondhand smoke exposure. To limit potential risk to the infant, breastfeeding mothers who use marijuana should be encouraged to abstain from or significantly reduce marijuana use.
American College of Obstetricians and Gynecologists (Marijuana Use During Pregnancy and Lactaqtion 2017)	There are insufficient data to evaluate the effects of marijuana use on infants during lactation and breastfeeding, and in the absence of such data, marijuana use is discouraged.
American Academy of Pediatrics (Policy Statement: breastfeeding and the use of human milk 2012; Ryan et al. 2018)	Present data are insufficient to assess the effects of exposure of infants to maternal marijuana use during breastfeeding. As a result, maternal marijuana use while breastfeeding is discouraged.
Academy of Breastfeeding Medicine (Reece-Stremtan and Marinelli 2015)	Breastfeeding mothers should be counseled to reduce or eliminate their use of marijuana to avoid exposing their infants to this substance and advised of the possible long-term neurobehavioral effects from continued use.

Considering the background of racial disparities (Racial and ethnic disparities in obstetrics and gynecology 2015; A tale of two countries: racially targeted arrests in the era of marijuana reform 2020) in maternal and infant health and relevant history of criminalization (A tale of two countries: racially targeted arrests in the era of marijuana reform 2020; Stone 2015) of substance use in pregnancy, we question whether practices regarding breastfeeding for those with marijuana use may exacerbate these harmful trends. We first perform a qualitative analysis of a random sample of prenatal records to illuminate instances of restricting breastfeeding for individuals with marijuana use in pregnancy. Then, we perform a scoping literature review of the evidence basis of current recommendations. Finally, we perform an empirically informed ethical analysis of practice regarding obligations to promote beneficence, autonomy, and justice.

## Methods

After Institutional Review Board approval, we performed a retrospective cross-sectional mixed-methods analysis. We reviewed prenatal and postpartum records from 150 randomly selected individuals who gave birth within an urban academic medical center hospital system during 2017 from the approach of an ethnography.

The study population consists of patients of varying race, age, employment, insurance, and educational status. Anyone without documentation from both prenatal and postpartum care visits was excluded, given our interest in the change in the language used by clinicians before and after delivery. This work is based on the narrative components of the electronic medical record, reviewing all prenatal, intrapartum, and postpartum notes for individuals who either reported marijuana use or tested positive for THC on a urinary toxicology screen. Epic software was used to search records systematically for the terms “THC” (delta-9-tetrahydrocannabinol) and “marijuana” and “cannabis.” All records with any narrative mentions were qualitatively reviewed for content, and all quotes related to the key terms were coded and included in the thematic analysis. Consistent with state policy, all pregnant patients were screened with urinary toxicology at prenatal care enrollment and upon admission for delivery. For the THC-exposed pregnancies (*n*=35), the entire prenatal record was reviewed and compared to a subset of patients without THC use until saturation was achieved. Data was not extracted, but reviewed initially as it appeared to clinicians in the relevant clinical workflow of Epic. This work, using traditional qualitative methods and grounded theory, was performed in the context of a broader study on language in prenatal records and thus breastfeeding content in the records of pregnancies with marijuana exposure was qualitatively compared to analogous content in demographically matched controls. We then performed a comprehensive scoping review of evidence regarding breastfeeding benefits for infants and women, followed by risks of breastmilk exposure to marijuana for infants relevant to a US population. PubMed, EMBASE, and Cochrane searches were performed and updated through 1/2021 using keywords such as “breastfeeding,” “marijuana,” “pregnancy,” “health benefits,” “infant abnormalities,” and “maternal wellbeing.” Relevant articles were reviewed, and additional sources were identified within references. The US health surveillance data, national policy, and professional society guidelines were also surveyed and critically reviewed. Finally, the practice of recommending against or restricting breastfeeding for women and infants with pregnancy marijuana exposure was analyzed vis-à-vis principles of beneficence, autonomy, and justice.

## Results

### Review of prenatal records

In our sample of patients (*n*=150), 35 had documented THC use during the index pregnancy. THC use was associated with younger mean age (27.5 ± 5.9 vs. 30 ± 6.1) and Black/African American race (80% versus 55%). Demographics of the sample are included in Table 2. By comparison to anecdotal content of prenatal records, which typically include at least some detailed discussion of feeding plans, details regarding breastfeeding counseling were relatively sparse. Each patient’s intended feeding plan was included in routine pregnancy care workflows with rare elaboration. Details on counseling content were included most often in circumstances where the patient was not planning to breastfeed despite no history of substance use or if the patient had a history of substance use and was planning to breastfeed. The most extensive documentation of antepartum breastfeeding counseling was by nursing at initial prenatal visits, and social workers after patients were referred for a positive toxicology screen. By comparison to those without THC use, patients with THC use in pregnancy were more likely to have discrepant feeding plans upon inpatient admission and hospital discharge, i.e., stated to admitting physician they were not planning to breastfeed, but were noted to be breastfeeding during postpartum care or vice-versa.

**Table 2:** Demographics

Characteristic		Total ***n***=150	Patients with THC use ***n***= 35	Patients without THC use ***n***= 115
Age	<18	2 (1.3%)	1 (2.9%)	1 (0.8%)
	18–24	38 (25.3%)	12 (34.3%)	26 (22.6%)
	25–29	39 (26.0%)	13 (37.1%)	26 (22.6%)
	30–34	33 (22.0%)	3 (8.6%)	30 (26.1%)
	>35	38 (25.3%)	6 (17.1%)	32 (27.8%)
Race	Black or African American	92 (61.3%)	28 (80%)	64 (55.7%)
	White or Caucasian	35 (28%)	7 (20%)	35 (30.4%)
	Asian	6 (4.0%)	0 (0%)	6 (5.2%)
	Other	11 (7.3%)	1 (2.9%)	10 (8.7%)
Feeding modality at time of discharge after delivery	Breast	105 (70%)	17 (48.6%)	88 (76.5%)
	Formula	34 (22.7%)	16 (45.7%)	18 (15.7%)
	Both	11(7.3%)	2 (5.7%)	9 (7.8%)

Qualitative analysis of content related to THC use and breastfeeding demonstrated themes of punitive language, emphasis on medicinal use, and restrictive approaches ranging from withholding of postpartum lactation support (e.g., breast pumps) to outright prohibition (e.g., “patient caught breastfeeding and instructed to stop.”). See Table 3 for representative quotes and analysis.

**Table 3:** Language in prenatal records related to marijuana use and breastfeeding: themes, representative quotes, and implications

Theme	Representative quotes—author/context	Implication
**Misinterpreting policy**	Pediatric NP in to see patient and inform her that she can no longer breastfeed due to positive tox for THC—postpartum nursing note	Evidence that policy to avoid marijuana use while breastfeeding has been misinterpreted to avoid breastfeeding.
	Advised patient that stopping smoking THC is beneficial for her and baby. Advised breastfeeding is not recommended for mothers who use THC … social work referral made—new OB intake visit nursing note	
	Reviewed decreasing THC use, pt is ‘attempting to do’… Pt aware she would not be able to breastfeed after delivery and SW would be involved if + urine tox at admission. Pt aware and will try to curb THC habit—antepartum social work note	
	Utox positive for THC: seen by SW postpartum. Avoid breastfeeding—postpartum physician note	
	Mom THC + on admit; express/discard until milk is safe 2 feed…pt aware of need to avoid THC exposure to infant—postpartum lactation consultant note	
**Medicinal use**	Patient states that she uses THC for her nausea and vomiting. States that she vomits up to 3 times every AM—nurse triage note	The risk-benefit discussion of marijuana use is nuanced considering secondary or tertiary underlying and related issues.
	Continues to admit to smoking marijuana, claiming that it helps her with increasing her appetite—antepartum social work note	
	Self-reports that she has experienced depression in the past, and has brought it up during past appointments with primary care doctors. She claims she never have been formally diagnosed or treated... Per the pt, she copes with her stress by smoking marijuana—antepartum social work note	
	Does admit to marijuana use secondary to headaches after her stroke—new OB provider note	
	Endorses self-medicating with marijuana daily, and states it is helpful in reducing her stress and anxiety—antepartum provider note	
	She is currently an everyday, several times a day marijuana smoker. She states it is the only thing that works to help with her nausea and her anxiety at this time. I reminded her that she has not been seen by a doctor yet to maybe prescribe her a legal and more healthy option to manage these 2 things—new OB nurse intake note	
	She reportedly smokes marijuana to help alleviate and cope with her current situational stressors (homeless, limited social support)—antepartum social work note	
**Punitive language**	Parent informed of need to discuss her history of substance abuse with Baltimore city CPS intake to determine a need for CPS intervention prior to maternal/newborn discharge. Parent is very anxious around pending CPS intervention and fearing removal postpartum social work note …no intervention would be required based on lack of a positive tox on delivery CPS intake worker She states no MJ use since leaving [addiction medicine program], but we don't have the utox results antepartum provider note	Despite not meeting legal requirements for Child Protective Services (CPS) referral, the patient was threatened with investigation and experienced anxiety surrounding the possibility of custody loss. This is an example of language that discredits the patient’s testimony.
	Discussed the implications of on-going substance abuse during pregnancy such as allegation of neglect leading to CPS intervention at delivery—antepartum note	This demonstrates power dynamic between patient and care system, highlighting punitive effect.
	Baby is NOT CLEARED until further notification from Baltimore City CPS. Social work will follow-up—postpartum social work note	
	Advised that she will possibly be sent to social work to address THC use during her next clinic visit—new OB nurse intake note	
**Withholding support**	Patient restricted from breastfeeding immediately following delivery due to urinary toxicology screen positive for THC. Postpartum note 3 months after delivery: she did not breastfeed after delivery but desires to start now. Pt asking for prescription meds to help produce lactation…discussed she may not be able to stimulate since she never breastfed	Limited breastfeeding support during labor/postpartum admission directly affected this patient’s ability to breastfeed her infant despite her expressed desire to do so.

### Literature review: weighing the evidence of risks and benefits

#### Impact on neonates

The endocannabinoid system is a network of retrograde neurotransmitters receptors throughout the brain and peripheral nervous system, implicated in a wide variety of cognitive and physiological processes, including mood, memory, appetite, pain-sensation, immunity, and fertility (Alger 2013). Considering health risks for infants, cannabinoids, like many other substances, cross the placenta (Grant et al. 2018). They are also present in breastmilk (Baker et al. 2018), with pharmacokinetics suggesting that an exclusively breastfed infant ingests approximately 2.5% of maternal THC dose (Baker et al. 2018). While some adverse effects of marijuana use on brain development are well-established (Volkow et al. 2014), no data exists evaluating neurodevelopmental outcomes beyond 1 year for infants exposed to marijuana exclusively through breastmilk, without exposure in utero (Reece-Stremtan and Marinelli 2015). For example, in one study (*n*=136), infants exposed to cannabis during breastfeeding found an associated decrease in the Bayley index of infant motor development at 1 year, though another (*n*=62) found no difference. In both cases, findings were confounded by THC use during pregnancy, and overall, the isolated effect of exposure through breastmilk on infants is unknown as there is significant overlap within utero exposure (Astley and Little 1990; Tennes et al. 1985). The demonstrated adverse effects of marijuana on child and adolescent neurodevelopment, with substantial impact on learning, memory, recall, IQ, and corresponding observed gray matter changes, raises legitimate concerns for newborn exposure: a period of unsurpassed neuroplasticity and development (Jacobus and Tapert 2014). Theoretical risks of neonatal THC exposure through breastmilk must therefore be weighed against the increased morbidity and mortality risks of foregoing breastfeeding for US infants.

A meta-analysis of 23 studies demonstrated that SIDS (sudden infant death syndrome), a leading cause of US infant mortality (Ely and Driscoll 2020), was twice as likely among formula-fed infants (McVea et al. 2000) corresponding with a 36% risk reduction with breastfeeding (Policy Statement: breastfeeding and the use of human milk 2012; Ip et al. 2007). Similarly, necrotizing enterocolitis (NEC) and sepsis have the highest incidence among formula-fed preterm infants and are reduced in NICU infants who receive human milk (McGuire and Anthony 2003). In a meta-analysis of four randomized controlled trials, breastfeeding decreased NEC by 58% (Victora et al. 2016), and in a large cohort of preterm infants (*n*=6198), breastfeeding significantly decreased urosepsis (OR 0.314, 95% CI 0.140–0.707, p < 0.009) (Levy et al. 2008).

Any breastfeeding was associated with a 64% reduction of nonspecific gastroenteritis, 23% reduction in otitis media, and dose-responsive 15–30% reduction in childhood obesity (Policy Statement: breastfeeding and the use of human milk 2012). Breastfeeding also decreases hospitalization for diarrhea (RR 0.28, 95% CI 0.14–0.54) (Horta and Victora 2013) and respiratory infections (RR 0.43, 95% CI 0.33–0.55) (Horta and Victora 2013) and has been associated with modest increases in IQ (Horta et al. 2015). To confer these benefits, the AAP recommends exclusive breastfeeding for the first 6 months of life followed by continued breastfeeding with the introduction of complementary foods for the first year and beyond, supported by the World Health Organization and the Institute of Medicine (Policy Statement: breastfeeding and the use of human milk 2012).

Forgoing breastfeeding is especially harmful to infants exposed to substances in utero by increasing the duration and severity of neonatal abstinence syndrome (Wu and Carre 2018) and increasing the likelihood of prolonged birth hospitalization and its downstream harms related to maternal-infant separation (Stuebe 2009), sensorimotor function, learning, and behavior (Maguire et al. 2016). Black and other socioeconomically marginalized infants are also more likely to suffer from virtually all conditions, and breastfeeding is considered protective for and thus may be differentially harmed by recommendations against breastfeeding.

### Impact on women

Meanwhile, breastfeeding presents even greater morbidity and mortality benefits for US women via reduction of postpartum blood loss and lower risk of breast, ovarian cancer or uterine cancer, cardiovascular disease, type 2 diabetes, and rheumatoid arthritis (Policy Statement: breastfeeding and the use of human milk 2012). According to the Women’s Health Initiative (*n*=139,681), breastfeeding for at least 12 months was associated with significant reductions in hypertension (OR=0.88), diabetes (OR= 0.88), hyperlipidemia (OR=0.81), and cardiovascular disease (OR=0.91) compared with women who never breastfeed.

This is a clinically significant reduction considering cardiovascular disease is the leading cause of mortality for U.S. women (Schwarz et al. 2009). Breast cancer is the most common cancer affecting US women. Breastfeeding for >12 months is associated with a 28% decrease in both breast and ovarian cancer (Policy Statement: breastfeeding and the use of human milk 2012). Furthermore, for every year of cumulative breastfeeding, the relative risk of breast cancer is decreased by 4.3% (95% CI 2.9–5.8, p<0.0001) (Collaborative Group on Hormonal Factors in Breast Cancer 2002). Lactational amenorrhea prevents 98% of pregnancies in the first 6 months after birth (Lactational Amenorrhea Method n.d.), with an associated reduction in risks of short interval pregnancies including low birthweight, preterm birth, precipitous labor, uterine rupture, gestational diabetes, and subsequent obesity (Hutcheon et al. 2019).

Recommending against breastfeeding must take into consideration the potential loss of maternal health benefits in addition to lost benefits for infant health and maternal-infant bonding. Though causality is unclear, there is a strong inverse association between breastfeeding and postpartum depression (Figueiredo et al. 2013), and breastfeeding is positively related to neurobiological, physical, and social dimensions of maternal-infant bonding (Liu et al. 2013). Additionally, children who were not breastfed face a greater risk for internalized behavioral problems, anxiety, and depression (Liu et al. 2013). Active maternal bonding while feeding is shown to result in lower rates of these behavioral problems as children age. Furthermore, DHA, an element of breastmilk, is also shown to play a role in decreasing the rates of developmental disorders such as ADHD (Stoner 2017).

### Clinical context

Our record review demonstrated frequent use of marijuana for the management of medical conditions, especially anxiety and depression (Stoner 2017). This is substantiated by a large study of a pregnant woman (*n* >25,000) that found maternal depression to be the highest risk factor for marijuana, alcohol, and tobacco use during pregnancy (Brown et al. 2019). High levels of maternal anxiety are associated with increased adverse outcomes ranging from impaired fetal neurobehavioral development, low birth weight, preterm labor, and preeclampsia (Dunkel Schetter and Tanner 2012; Kinsella and Monk 2009). Chronic stress is shown to decrease the brain’s production of endocannabinoids. Introducing cannabis into the system aids in the restoration of normal endocannabinoid levels and therefore alleviates symptoms of depression and depression-like behavior. By contrast, THC and CBD have demonstrated efficacy in mitigating anxiety, PTSD, and other anxiety-related disorders (Blessing et al. 2015) which are often especially acute while caring for a newborn. Likewise, the release of oxytocin during breastfeeding has a soothing, analgesic effect on mother and baby (Infant and young child feeding: model chapter for textbooks for medical students and allied health professionals 2009).

The socio-behavioral costs of not breastfeeding may also be significant and disproportionately harm low-income and minority women. Stigmatization (Bresnahan et al. 2019) and social exclusion (e.g., from Facebook groups) of formula feeding mothers may exacerbate harm for those with coinciding mood disorders. Though not straightforward, the financial ramifications of avoiding breastfeeding can be significant for individual families and on societal levels. Loss of WIC benefits at 6 vs 12 months because of formula use may impact the mother, infant, and other siblings. Costs of care for infants with more frequent mild and severe infections, in addition to the lost wages of their mothers and other care providers may be substantial. Over time, lost lifetime wages for women affected by cardiovascular disease and cancer also impact financial wellbeing.

## Discussion: empirically informed ethical analysis

The benefits of breastfeeding for infants and women are well-established. The Academy of Breastfeeding Medicine acknowledges that current evidence is “not strong enough” to recommend against breastfeeding for women who use marijuana (Reece-Stremtan and Marinelli 2015), and therefore, maternal marijuana use is not considered a categorical contraindication to breastfeeding (Ryan et al. 2018), consistent with CDC, ACOG, and AAP guidance (Table 1).

Exposure to marijuana during pregnancy confers significant potential harm to infants. Studies elucidating the impact of marijuana exposure through breastmilk are limited and difficult to interpret. Thus, based on reasonable theoretical harm, national guidelines (Table 1) encourage breastfeeding and discourage marijuana use.

### Misinterpreting policy distorts beneficence

In practice, as seen in the Misinterpreting Policy section of Table 3, women who use marijuana may be told to avoid breastfeeding: a fallacy of assuming the converse holds true. There is no evidence base to discourage breastfeeding and doing so outweighs a known harm with a theoretical one. The assertion that women who use marijuana should not breastfeed incorrectly asserts that the harm to infants from marijuana exposure in breastmilk outweighs the many known and well-established harms of not breastfeeding for both neonates and mothers.

Discouraging breastfeeding, suggesting that breastfeeding is “not recommended,” or indicating that breastfeeding is contraindicated with marijuana use are examples of the misinterpretation of these guidelines. Even when correctly interpreted, the current national guidelines do not consider the medicinal use of marijuana. Despite the perception of marijuana use as recreational, the most common reasons reported for marijuana use during pregnancy among patients at a Canadian tertiary care center were anxiety (33.3%), nausea/vomiting (22.2%), and sleep (22.2%) (Manning and Drover 2020). Our review of records similarly demonstrates non-recreational use of marijuana for alleviating nausea, anxiety, depression, headaches, and low appetite as seen in Table 3. The medicinal use of cannabinoids is increasing and relevant for risk-benefit analysis. Due to the lack of existing evidence documenting its direct harm, marijuana is not a contraindication to breastfeeding. If marijuana is being used to mitigate anxiety or insomnia, is restricting its use among breastfeeding women an appropriate recommendation? The American Academy of Pediatrics recommends initiation of breastfeeding with the use of benzodiazepines, another medication used to treat anxiety, that crosses the placenta, is present in breastmilk, and has limited evidence to support adverse effects on infants (Kelly et al. 2012). The AAP similarly acknowledges the opportunity for limiting risk to infants by limiting exposure but does not recommend against benzodiazepine use.

Misinterpretation of the national guidelines is not in line with beneficence. Furthermore, those infants exposed to marijuana during pregnancy may be at disproportionately increased risk of outcomes positively affected by breastfeeding and depriving them of breastmilk may compound harm. Any barriers to accessing human milk may be considered harmful in the absence of evidence that risks of secondary marijuana exposure exceed known benefits (Policy Statement: breastfeeding and the use of human milk 2012).

### “She’s not allowed:” compromising autonomy

In a recent study evaluating providers’ response to self-disclosed cannabis use during a prenatal visit, 90 patients disclosed marijuana use and in 48% of these visits, providers did not offer counseling or information related to this disclosure. Among those who received counseling, Black women were at a 10-fold risk of punitive counseling vs. White counterparts (Holland et al. 2016a). Furthermore, when counseling was offered, 70% of the time was dedicated to punitive ramifications including legal implications and involvement of CPS (Holland et al. 2016a). Finally, a series of semi-structured interviews with obstetricians demonstrated a lack of familiarity with the risks of marijuana use in pregnancy and counseling strategies that were punitive in nature (Holland et al. 2016b). Our qualitative analysis similarly revealed a lack of counseling regarding feeding choices for outpatients. Furthermore, counseling was included most often when a patient was planning to breastfeed and had a history of substance use, suggestive of general practices of documenting patient plans and relevant counseling when they diverge from clinician standard of care recommendations. A perception that a patient is “not allowed” to breastfeed while inpatient further undermines the ability to make an informed, shared decision.

Critically, immediate postpartum breastfeeding initiation and education are associated with breastfeeding at discharge from the hospital and continuation at 6 months postpartum (Cohen et al. 2018). Delayed initiation compromises milk supply and infant receptivity, prompting professional strong recommendations to s mothers to breastfeed within the first hour after birth: the “golden hour.” Though patients are theoretically able to initiate breastfeeding once they leave the hospital, the first hour and days of life are a singular opportunity to establish effective lactation. Withholding critical support during postpartum hospitalization further compromises autonomy, particularly given concurrent physical, mental, and emotional vulnerability as a captive audience. The patient with THC use in pregnancy was more likely to have discrepant feeding plans upon inpatient admission and hospital discharge. This dissonance was also suggestive of tension between patients and providers regarding infant feeding in the setting of in utero marijuana exposure. Our prenatal record analysis is consistent with the observed refusal of lactation consultants, breast pumps, and other essential breastfeeding supplies to postpartum patients for whom breastfeeding was not recommended as a result of drug screening results. Furthermore, women and infants are subjected to various degrees of state-dependent mandatory drug testing and reporting. In states where screening is performed, frequently coinciding with states where marijuana legalization has stalled, the presence of marijuana on mother or infant screens during delivery admission triggers reporting to state Child Protective Services (CPS). In these states, CPS reflexively performs a hospital evaluation and home visits for any substance-exposed neonate. Reporting requirements are typically insensitive to frequency, timing, duration, or reason for marijuana use. These threatening policies also discourage substance-using women from seeking medical treatment during their pregnancies (Stone 2015), intensifying the danger for both mother and child.

In 18 states, drug use during pregnancy is legally considered child abuse (How States Handle Drug Use During Pregnancy n.d.). Awareness of the punitive environment further threatens patient autonomy, and patients’ concerns about legal consequences were demonstrated in the qualitative analysis (Table 3). Depending on how these issues are framed, providers may inadvertently exacerbate patient anxiety. Ultimately, free and informed decisions about the risks and benefits of the infant feeding methods are not possible when one fears that breastfeeding may jeopardize child custody.

### Disparities in breastfeeding and criminalization: exacerbating injustice

Low-income and minority women have lower healthcare literacy, decreased access to care, short interpregnancy intervals, and more unintended pregnancies at younger ages (Thoma et al. 2019), all factors associated with marijuana use in pregnancy (Ryan et al. 2018). These women are at increased risk for preterm birth (Thoma et al. 2019), infant mortality (Ely and Driscoll 2020), SIDS (Sudden unexpected infant death and sudden infant death syndrome n.d.), diabetes (Racial and ethnic disparities in obstetrics and gynecology 2015), obesity (Breastfeeding in underserved women: increasing initiation and continuation of breastfeeding 2013), cardiovascular disease (Breastfeeding in underserved women: increasing initiation and continuation of breastfeeding 2013), and breast cancer (Racial and ethnic disparities in obstetrics and gynecology 2015). The national average rate of breastfeeding initiation is 75%; however, significant disparities exist based on income, age, and ethnicity, with the lowest rates (30%) seen among young, low-income Black mothers (Policy Statement: breastfeeding and the use of human milk 2012). ACOG acknowledges both the additional barriers that preclude minority women from breastfeeding as well as the disproportionate harm and socioeconomic burden they may experience without breastfeeding (Breastfeeding in underserved women: increasing initiation and continuation of breastfeeding 2013).

Additionally, racial disparities in the criminalization of substance use in general, including marijuana (A tale of two countries: racially targeted arrests in the era of marijuana reform 2020), highlight the punitive harm of CPS involvement. Despite comparable usage, Black race was associated with a 3–10 times greater likelihood of arrest for marijuana possession (A tale of two countries: racially targeted arrests in the era of marijuana reform 2020). This practice is upheld within the confines of obstetric care as is evidenced by a nearly 10-fold increase in reporting of Black women’s substance use during pregnancy, though the actual rate of substance use was similar (Chasnoff et al. 1990).

Finally, disparities in breastfeeding initiation (Breastfeeding in underserved women: increasing initiation and continuation of breastfeeding 2013) are attributed to barriers disproportionately experienced by Black women, including inadequate information from providers and a lack of lactation support (Cohen et al. 2018), both of which may be affected by current practices regarding marijuana and breastfeeding. The underlying disparities that exist in breastfeeding initiation and marijuana criminalization may be exacerbated by restrictive interpretations of national policy.

## Conclusion

Current guidelines may prevent vulnerable women and infants from breastfeeding despite limited evidence of harms and well-established benefits, contradicting beneficence, compromising autonomy, and exacerbating disparities. We provide evidence of common misinterpretation of recommendations regarding minimizing or avoiding marijuana use during breastfeeding as a contraindication to breastfeeding when marijuana use is present. Though our data are limited to a single setting, our clinical experience and ongoing qualitative interviews of birth providers demonstrate a range of practices wherein restrictive approaches often apply. We find that discouraging breastfeeding among women who use marijuana is likely to present greater risks than benefits to mother-infant dyads. Though women have a right to make informed decisions about infant feeding, their autonomy may be effectively curtailed by withholding essential lactation support immediately postpartum and punitive threats of child welfare investigation. Finally, a restrictive interpretation of breastfeeding policy may contribute to existing racial disparities in breastfeeding initiation and women and children’s related health outcomes. We advocate for further work to expand the content and nuance of guidelines, with attention to the risks of punitive, stigmatizing, and biased contexts with unintended consequences, and the duty to provide support for safe, shared decision making.

## Data Availability

All data generated or analyzed during this study are included in this article.

## Abbreviations

THC: Tetrahydrocannabinol
IRB: Institutional Review Board
IQ: Intelligence quotient
SIDS: Sudden infant death syndrome
NICU: Neonatal intensive care unit
DHA: Docosahexaenoic acid
ADHD: Attention-deficit/hyperactivity disorder
CBD: Cannabidiol
PTSD: Post-traumatic stress disorder
FDA: U.S. Food and Drug Administration
SSRI: Selective serotonin reuptake inhibitors
WIC: Women, infants, and children
CDC: Centers for Disease Control and Prevention
ACOG: American College of Obstetricians and Gynecologists
CPS: Child Protective Services
Pt: Patient
